# Krukenberg Tumor Related to Gallbladder Cancer in a Young Woman: A Case Report and Review of the Literature

**DOI:** 10.3390/jpm13060957

**Published:** 2023-06-06

**Authors:** Giulia Grizzi, Michele Ghidini, Margherita Ratti, Marianna D’Ercole, Giulia Tanzi, Annalisa Abbiati, Andrea Celotti, Daniele Spada, Gian Luca Baiocchi, Maria Bonomi

**Affiliations:** 1 Oncology Unit, ASST Cremona, 26100 Cremona, Italy; 2 Oncology Unit, Fondazione IRCCS Ca’ Granda Ospedale Maggiore Policlinico, 20122 Milan, Italy; 3 Department of Pathology, ASST Cremona, 26100 Cremona, Italy; 4 Gynecology Unit, ASST Cremona, 26100 Cremona, Italy; 5 General Surgery Unit, ASST Cremona, 26100 Cremona, Italy

**Keywords:** Krukenberg tumor, gallbladder cancer, ovarian mass, biliary tract cancer, chemotherapy

## Abstract

A gallbladder tumor is a rare condition, which usually spreads to the liver, lymph nodes, and other organs. A Krukenberg tumor, derived from the biliary tract and gallbladder cancers (GBCs), is an uncommon finding in routine clinical practice. Here, a case of a young woman with a Krukenberg tumor related to a previous diagnosis of GBC is reported. Differential diagnosis of an ovarian malignant lesion is challenging for both clinicians and pathologists. In order to provide a proper diagnosis, integrated multidisciplinary management is essential. The occurrence of Krukenberg tumors should be evaluated in the management of GBC, even if this is rare in clinical practice.

## 1. Introduction

A Krukenberg tumor is a secondary lesion of the ovary from a mucin-rich signet-ring adenocarcinoma that most commonly arises from gastric (41%) and colorectal cancers (49%) [1,2,3]. Less frequently it can derive from breast, appendix, pancreatic, small intestine, urinary bladder, biliary tract, ampulla of Vater, or uterine cervix cancers [1]. Given their metastatic nature, most of these tumors are bilateral (80%) [4]. They can present as a synchronous lesion (i.e., within three months of the primary tumor’s diagnosis) or as a metachronous lesion, even years after the primary tumor has been treated. Overall, an ovarian lesion, which is suspected to be malignant, will be determined to be metastatic in 5–25% of all ovarian cancers [4,5]. The differential diagnosis of an ovarian malignant lesion (primary vs. metastatic) represents a challenge for both clinicians and pathologists. In order to identify the correct diagnosis and prognosis, integrated multidisciplinary management is crucial.

Gallbladder cancer (GBC) usually spreads to the liver, lymph nodes, and other sites of the gastrointestinal tract by direct invasion. A Krukenberg tumor derived from the gallbladder and extrahepatic biliary malignancies is a rare condition, which significantly worsens the prognosis [3,6]. Radical surgery is the only treatment that can cure GBC completely [7,8]. After surgery, adjuvant capecitabine-based chemotherapy (CT) and radiotherapy (RT) are recommended options, in particular in the presence of risk factors of early relapse such as R1 resection or nodal-positive disease [9,10]. Despite adjuvant treatment, the rates of systemic recurrence are high, which requires the activation of first-line palliative treatment. A gemcitabine–cisplatin-based regimen has been the standard treatment in the first-line setting for more than ten years. In the last few years, recent advances have shown interesting results in associating the inhibitor of the anti-programmed death-ligand 1 (PD-L1) durvalumab with the aforementioned CT [11]. Meanwhile, a targeted therapy approach, based on molecular profiling, has enlarged the landscape of care in the treatment of advanced GBC (i.e., HER2 overexpression/amplification). Disappointingly, unlike intrahepatic cholangiocarcinoma, FGFR2 translocation as well as IDH1/2 mutation, which are the target of specific drugs, are infrequent in GBC (FGFR2- translocation 3% and IDH1/2 mutation 2%) [12]. Furthermore, the targetable BRAF V600e mutation is restricted to intrahepatic BTC [13]. After progression on first-line treatment and in the absence of target mutations, little evidence is available to suggest that the 5-fluorouracil-oxaliplatin CT doublet should be considered as the preferred second-line regimen [14].

We present an uncommon case of a woman with a Krukenberg tumor related to previous GBC.

## 2. Case Report

A 52-year-old Caucasian woman, with no comorbidities in her history was admitted to the surgery department with clinical, laboratory, and radiological evidence of acute cholecystitis. A laparoscopic cholecystectomy plus lymphadenectomy was performed. The histological examination revealed an incidental diagnosis of fundus-body adenocarcinoma of the gallbladder, with a clear cell component, pT2b, Ki-67 80%, metastasis in one out of two removed lymph nodes, and mismatch repair proficient (pMMR). Therefore, the patient underwent a second laparoscopic surgery to perform hepatic bed resection and extended lymphadenectomy (1 out of 19 lymph nodes positive); the results of peritoneal washing were negative for malignant tumor cells.

**Figure 1:**
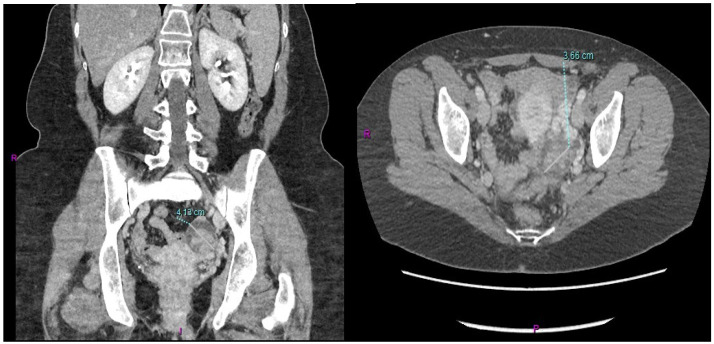
Metastatic involvement of the left ovary derived from the previous GBC.

After conjunct oncological and RT assessment, the patient received adjuvant combined CT-RT treatment for a total of 6 months (four cycles of adjuvant capecitabine before and after adjuvant RT with capecitabine as a sensitizer). The CT scan performed a month after the end of adjuvant treatment showed the presence of a 3.6 cm left ovarian mass in the absence of other signs of local or distant relapse of the previous GBC (Figure 1).

A gynecological examination was performed, which confirmed the compound left ovarian lesion. Markers including carcinoembryonic antigen (CEA), cancer antigen (Ca) 19.9, Ca125, alpha-fetoprotein (AFP), and beta-human chorionic gonadotropin (b-HCG), were normal. Due to the clinical suggestion of a primary ovarian tumor, the patient underwent laparoscopic bilateral salpingo-oophorectomy and endometrial biopsies. However, the extemporaneous histological examination was compatible with the ovarian metastasis of the previous GBC. The definitive left ovary histology confirmed the presence of a secondary adenocarcinoma morpho-phenotypically similar to the tumor in anamnesis, while the right ovary and endometrial biopsies were negative. The results of peritoneal washing were positive for malignant tumor cells.

**Figure 2:**
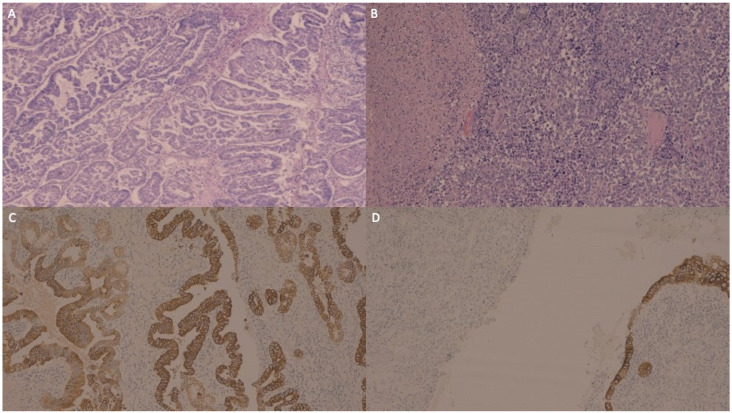
The carcinoma of the gallbladder was composed of a moderately differentiated component presenting complex glandular architecture (**A**) and a poorly differentiated component with solid growth and foci of necrosis (**B**). The moderately differentiated component exhibited diffuse and intense immunoreactivity for cytokeratin AE1/AE3 (**C**), focal positivity for cytokeratin 8/18, and only focal and scant positivity for cytokeratin 19 and cytokeratin 7 (not shown), while the poorly differentiated component was largely negative for the cited markers (in (**D**), the poorly differentiated gallbladder carcinoma stains were negative for cytokeratin AE1/AE3, while the normal gallbladder columnar epithelium is shown in the bottom right corner as the internal positive control).

Microscopic examination showed that the GBC was composed of a moderately and a poorly differentiated component (Figure 2A,B). The immunohistochemistry (IHC) of the moderately differentiated component exhibited diffuse and intense immunoreactivity for cytokeratin (CK) AE1/AE3 (Figure 2C), focal positivity for CK 8/18 and only focal positivity for CK19 and CK7. The poorly differentiated component was largely negative for the cited markers (Figure 2D).

**Figure 3:**
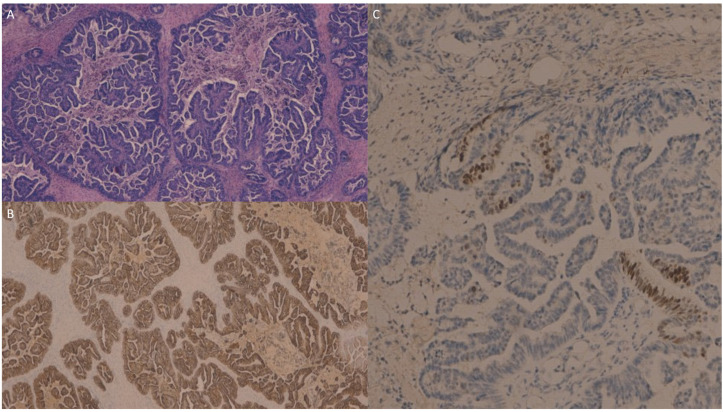
The ovarian metastasis from gallbladder carcinoma (**A**) was intensely and diffusely immunoreactive for cytokeratin 7 (**B**) and showed focal positivity for CDX2 (**C**), while it was negative for PAX8 (not shown).

The ovarian metastasis from the gallbladder carcinoma (Figure 3A) was intensely and diffusely immunoreactive for CK7 (Figure 3B) with a focal positivity for CDX2 (Figure 3C), while it was negative for PAX8. 

**Figure 4:**
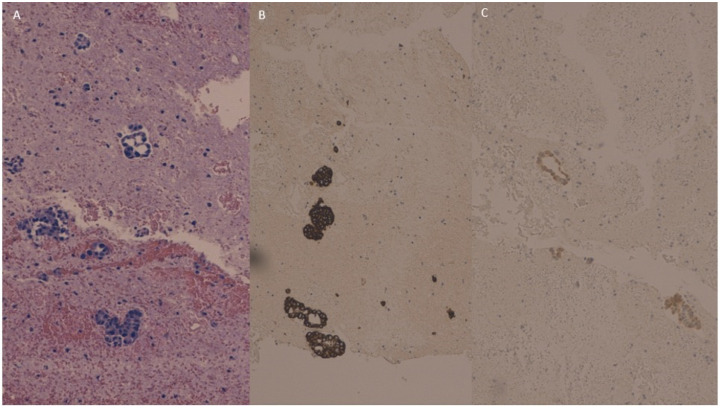
Peritoneal washing cell block showing neoplastic glandular and micropapillary structures (**A**) which were intensely positive for cytokeratin 7 (**B**) and weakly positive for Cytokeratin 20 (**C**).

The peritoneal washing cell block showed neoplastic glandular and micropapillary structures (Figure 4A) which were intensely and diffusely positive for CK 7 (Figure 4B) and weakly positive for CK 20 (Figure 4C).

The post-operative CT scan showed a 10 mm nodule on the right abdominal side compatible with subcutaneous metastases and mild peritoneal involvement. No first-line clinical trials were available at that time. Therefore, on August 2022, the patient started first-line standard CT with cisplatin and gemcitabine (durvalumab was not yet approved in Italy, nor was the patient a candidate for the expanded access program because their systemic relapse had occurred before 6 months after the completion of adjuvant therapy had passed). The CT scan assessment conducted after four cycles, showed a stable disease. The patient received a total of eight cycles of the cisplatin–gemcitabine first-line CT doublet. The treatment was well tolerated with no major hematological or non-hematological toxicities.

Moreover, during first-line treatment, in order to plan subsequent lines of therapy, a Foundation One CDx biopsy on the ovary tissue was performed. The test evidenced a genomic signature with a microsatellite stable status and a tumor mutational burden with four mutations per megabase. No relevant genes with targetable alterations were identified. The gene alterations detected were the sequent: CTNNB1 (S37F), MTAP (loss), STK11 (H174R), CDKN2A/B (CDKN2A loss and CDKN2B loss), SMAD4 (A418fs*6), SMARCA4 (R1192C), and XPO1 (E571K–subclonal), but no clinical trials were available in our country.

Unfortunately, the CT scan performed at the end of the eight cycle evidenced worsening of the disease with the appearance of lymph node metastases on the celiac axis, an increase in ascites, and stable size of the subcutaneous nodule in the right abdominal side. According to international guidelines, the patient started second-line doublet CT with 5-fluorouracil and oxaliplatin which is ongoing at present.

## 3. Discussion

GBC is a rare tumor whose early diagnosis is challenging because the symptoms are similar to those of benign diseases (e.g., cholecystitis, polyps, and adenomyomatosis) and to those of other hepatobiliary cancers such as intrahepatic cholangiocarcinoma and hepatocellular carcinoma. Most cases of GBC are incidentally diagnosed during pathological examination after elective or emergency cholecystectomy. Up to now, surgery is the only treatment that can completely cure GBC and in general BTC. Cholecystectomy is considered adequate for T1a GBC resulting in high 5-year survival rates [8], while extended resection (including secondary oncologic resections of incidental GBC) is recommended for tumors of stage T1b or higher [7]. In T1b or higher GBC, a locoregional lymphadenectomy is mandatory because lymph node spread is one of the most important prognostic factors in the resected disease [15].

Unfortunately, the 3-year recurrence rate after radical surgery is up to 80% [16,17], underling the relevance of adjuvant therapy. In particular, the two high-risk populations that benefit most from an adjuvant CT are patients with nodal-positive disease and those with an R1 resection [10,11]. At present, three randomized controlled studies (PRODIGE-12 [18], BCAT [19], and BILCAP [20]) have not shown significant improvement in adjuvant CT outcomes. However, in a prespecified per protocol analysis of the BILCAP study, median overall survival (OS) was significantly higher with capecitabine (3-weekly, 8 cycles) compared with observation (53 months versus 36 months, respectively; hazard ratio [HR] 0.75, 95% confidence interval [CI], 0.58–0.97, *p* = 0.028). Moreover, in the intention-to-treat (ITT) population, relapse-free survival (RFS) with capecitabine was higher during the first 24 months [20]. Despite limited evidence, adjuvant CT with capecitabine should be taken into consideration for patients with GBC after radical resection, especially in case of risk factors of disease recurrence (R1, nodal-positive disease, and grade 3–4). A few pieces of data supporting adjuvant radiotherapy are available, mostly derived from retrospective studies. In the phase II SWOG S0809 trial, the primary objective of the study (a 2-year survival rate >45%) was met, suggesting that RT (with capecitabine as a sensitizer) after completion of adjuvant CT might be considered in selected patients such as those with R1 resection or nodal-positive GBC [21].

In advanced GBC, CT with cisplatin and gemcitabine is the current standard of care for first-line CT. For more than a decade, the phase 3 ABC-02 trial established the superiority, in terms of OS and PFS, of the cisplatin–gemcitabine doublet over gemcitabine alone in patients with BTC and in the 149 (36.3%) patients with GBC who were included in the study [22]. Recently, the phase 3 TOPAZ-1 trial randomized patients with advanced biliary tract cancer to receive durvalumab or a placebo in combination with gemcitabine plus cisplatin for up to eight cycles, followed by durvalumab/placebo monotherapy until progression or unacceptable toxicity. The primary end-point of the study was OS, while the secondary end-points included progression-free survival (PFS), objective response rate (ORR), and safety. Globally, 685 patients were included, and the HR for OS was 0.80 (95% CI, 0.66 to 0.97; *p* = 0.021). The estimated 24-month OS rate was 24.9% (95% CI, 17.9 to 32.5) for durvalumab and 10.4% (95% CI, 4.7 to 18.8) for the placebo. As the secondary end-points, the HR for PFS was 0.75 (95% CI, 0.63 to 0.89; *p* = 0.001) and the ORR was 26.7% with durvalumab and 18.7% with the placebo. The incidences of grade 3 or 4 adverse events was similar for the two treatments (75.7% and 77.8% with durvalumab and placebo, respectively). In particular, 171 (25%) patients affected by GBC were included, and no differences in terms of PFS and OS were observed regarding the primary tumor site. Based on this evidence, the combination of cisplatin–gemcitabine plus durvalumab is considered as the new first-line treatment standard of care for advanced GBC [11].

In order to offer personalized treatment to each patient, a molecular analysis should be considered during or after the progression of first-line CT. The most common genetic alterations in GBC are human epidermal growth factor receptor 2 (HER2) overexpression or amplification [23,24], high expression of vascular endothelial growth factor (VEGF), [25], epidermal growth factor receptor (EGFR) overexpression [26], mitogen-activated protein kinase (MAPK) pathway alteration [27], and C-mesenchymal–epithelial transition factor (c-MET) overexpression [28]. Although preliminary, the most consistent data concern anti-HER2 targeted drugs such as the combination of pertuzumab plus trastuzumab and the pan-HER inhibitor neratinib. The multicenter phase 2a MyPathway basket trial is evaluating the effect of a combined regimen with pertuzumab plus trastuzumab in patients with BTC and HER2 amplification or overexpression. The preliminary data have shown a promising ORR of 23% in the first 39 recruited patients, leading to a partial response (PR) in 9 patients [29]. In the phase 2 SUMMIT basket trial, the treatment outcomes of 25 patients in the biliary tract cancer cohort, including 10 affected by GBC (40%), were presented. Among the 10 pretreated HER2-positive GBC patients, 3 patients showed a PR after receiving neratinib, with a preliminary PFS of 3.7 months and an OS of 9.8 months [30]. Moreover, in the phase II HERB trial (JMA-IIA00423), the anti-HER2 antibody–drug conjugate trastuzumab–deruxtecan showed an interesting ORR (36.4%) and disease control rate (81.8%) in the 22 BTC patients enrolled, including 11 with GBC [31]. Unfortunately, unlike intrahepatic cholangiocarcinoma, FGFR2 translocations, which can be targeted by FGFR inhibitors (pemigatinib, infigratinib, and erdafitinib), as well as IDH1/2 mutations (targetable with the IDH inhibitor ivosidenib) are rare in GBC (FGFR2 translocations 3%; IDH1/2 mutations 2%) [12]. Furthermore, the well-targetable BRAF V600e mutation seems to be restricted to intrahepatic BTC [13].

After disease progression during first-line CT and in the absence of target mutations, a few pieces of evidence are available for second-line CT. In the multicenter phase 3 ABC-06 trial, patients with locally advanced or metastatic BTC were randomized to receive FOLFOX plus active symptoms control (ASC) or ASC alone. The primary end point of the study was OS in the ITT. In total, 162 patients were enrolled of whom 34 were affected by GBC. The primary end point of the study was met with a significantly longer median OS of 6.2 months (95% CI 5.4–7.6) in the ASC plus FOLFOX group versus 5.3 months (4.1–5.8) in the ASC alone group (HR 0.69 [95% CI 0.50–0.97]; *p* = 0.031). At 6 months, the OS rate in the ASC alone group was 35.5% (95% CI 25.2–46.0) compared with 50.6% (39.3–60.9) in the ASC plus FOLFOX group, and at 12 months, the OS rate was 11.4% (5.6–19.5) versus 25.9% (17.0–35.8), respectively [14].

A Krukenberg tumor (whose name is derived from the German physician Friedrich Ernst Krukenberg) is a malignancy of the ovary that metastasizes from a primary cancer site, typically from the gastrointestinal tract and in particular from gastric cancer. Ovarian spread is an extremely rare occurrence in GBC, while the most common metastatic sites are the liver (76–86%) and lymph nodes (60%) [32].

In clinical practice, the differential diagnosis of an ovarian mass is complicated, and 4.7% of ovarian cancers are metastatic [3]. Most of these derive from primary tumors of the gastrointestinal tract, in particular from colorectal (49%) and gastric (41%) tumors, while only 1.5% derive from gallbladder and biliary tract cancers [3,6]. The metastatic spread pathway to the ovaries from the gastrointestinal–biliary tract is still unknown. Laterality of the ovary involved in metastasis is not associated with the site of the primary tumor, while lymph nodal metastases are frequently found in Krukenberg tumors [4]. This suggests that metastases follow a lymphatic path and that the ovaries are involved as a retroperitoneal organ.

Ovarian malignancies often pose a differential diagnostic problem for both clinicians and pathologists, but the distinction between primary versus secondary tumors is crucial in order to identify the correct treatment and prognosis. In general, ovarian metastases are typically smaller than 10 cm in diameter and usually contain cysts [33]. Bilaterally ovarian malignancies are common in metastatic cancer, and they were reported to occur in more than 80% of all Krukenberg tumors [4]. Therefore, the high rate of bilaterality, surface involvement by tumor cells, multinodular growth, a size <10 cm, an infiltrative and nodular pattern of invasion, and signet ring cells are the most helpful features suggesting the metastatic nature of an ovarian mass [34].

Combined to clinical examination, radiological exams are useful for a better definition of the disease, suggesting the importance of a complete preoperative evaluation and avoiding unnecessary surgery for metastatic ovarian lesions. A retrospective study aimed to assess the diagnostic value of staging laparoscopy in identifying disseminated disease in primary GBC (pGBC) and in incidentally GBC (iGBC) planned for (re)-resection. In total, 290 patients were enrolled, 183 with pGBC and 107 with iGBC. The staging laparoscopy was performed in 40 pGBC patients and 7 iGBC patients. A disseminated disease was found in eight and one patients, respectively. Based on the outcome of this study, in the pGBC patients, staging laparoscopy might identify a disseminated disease in up to 20% of patients and should be part of standard management because its yield is significant with limited investment of time and medical resources. As iGBC is often diagnosed at an earlier stage, the importance of staging laparoscopy seems moderate and should be considered in patients with a high risk of advanced disease such as in those with cholecystitis or initial R1/R2 resection [35].

The role of IHC in identifying primary and secondary ovarian cancer is useful but has some limits. First of all, there are no specific antibodies that can distinguish these tumors with absolute certainty. CK7 and CK20 are commonly used markers in ovarian tumors [36,37]. Primary ovarian cancers are almost always positive for CK7 but are typically negative for CK20. CDX2 is usually negative for primary ovarian malignancies, whereas it is positive for ovarian metastases from the colon or GBC [38].

Complementary to the macroscopic and histological characteristics, the age of the patient represents an important feature in differentiating primary from metastatic ovarian cancers. A previous study showed that only 9.1% of ovarian metastases occur in women younger than 50 years, while in the group with primary tumors, 49% of patients were younger than 50 years [39].

Up to now, there is no consensus on the treatment approach of ovarian metastasis particularly for Krukenberg tumors derived by GBC. To improve survival outcomes, cytoreductive surgery (in the absence of a massive spread of the disease or pleural effusion) and palliative CT (plus immunotherapy in the first-line setting, if available) or targeted therapy in the presence of molecular alterations are recommended options.

## 4. Conclusions

The evidence of an ovary malignancy often poses a differential diagnosis challenge for both clinicians and pathologists. While some secondary lesions are clearly metastatic, others may be confused with ovarian cancer in particular when the primary tumor is occult at diagnosis.

GBC is a rare cancer that can be diagnosed incidentally after elective or emergency cholecystectomy as in the case report we have described. Up to now, surgical resection is the only curative therapy option. After surgery, only a subgroup of high-risk patients (R1, N+, and grade 3–4) might benefit from adjuvant treatment. Unfortunately, after surgery and adjuvant chemo-radiotherapy, recurrence rates are high and require systemic treatment. As first-line treatment, a palliative CT regimen with cisplatin and gemcitabine has been the standard of care for more than a decade. Recently, the addiction of the anti-PD-L1 durvalumab to the aforementioned CT has improved OS outcomes, with it becoming the new standard of care for unresectable GBC. After the failure of upfront therapy, the FOLFOX doublet is the only established treatment option for second-line therapy. With the rising importance of tailored therapy approaches based on molecular profiling, the unique molecular alterations of GBC underline the opportunity to improve patients’ outcomes by molecularly targeted therapies.

Metastatic spread of GBC usually involves the lymph nodes and liver, while involvement of the ovaries is extremely rare. However, the occurrence of Krukenberg tumors should be considered in the management of GBC, whilst being aware that this is a rare condition in clinical practice. A correct clinical–pathological diagnosis is fundamental in order to identify the right treatment and prognosis for every single patient.

## Data Availability

Data sharing is not applicable.
